# Lower limb orthopaedic surgery results in changes to coagulation and non-specific inflammatory biomarkers, including selective clinical outcome measures

**DOI:** 10.1186/2047-783X-18-40

**Published:** 2013-11-09

**Authors:** Stephen F Hughes, Beverly D Hendricks, David R Edwards, Salah S Bastawrous, Jim F Middleton

**Affiliations:** 1 Department of Biological Sciences, University of Chester, Chester, UK; 2 Leopold Muller Arthritis Research Centre, RJAH Orthopaedic Hospital, Medical School, Keele University, Keele, UK; 3 Haematology Department, BCUHB, Glan Clwyd Hospital, North Wales, UK; 4 Haematology Department, BCUHB, Gwynedd Hospital, North Wales, UK; 5 Orthopaedics Department, BCUHB, Glan Clwyd Hospital, North Wales, UK; 6 Faculty of Medicine and Dentistry, School of Oral and Dental Science, University of Bristol, Bristol, UK

**Keywords:** Coagulation, Inflammation, Orthopaedic surgery

## Abstract

**Background:**

With an aging society and raised expectations, joint replacement surgery is likely to increase significantly in the future. The development of postoperative complications following joint replacement surgery (for example, infection, systemic inflammatory response syndrome and deep vein thrombosis) is also likely to increase. Despite considerable progress in orthopaedic surgery, comparing a range of biological markers with the ultimate aim of monitoring or predicting postoperative complications has not yet been extensively researched. The aim of this clinical pilot study was to test the hypothesis that lower limb orthopaedic surgery results in changes to coagulation, non-specific markers of inflammation (primary objective) and selective clinical outcome measures (secondary objective).

**Methods:**

Test subjects were scheduled for elective total hip replacement (THR) or total knee replacement (TKR) orthopaedic surgery due to osteoarthritis (*n* = 10). Platelet counts and D-dimer concentrations were measured to assess any changes to coagulation function. C-reactive protein (CRP) and erythrocyte sedimentation rate (ESR) were measured as markers of non-specific inflammation. Patients were monitored regularly to assess for any signs of postoperative complications, including blood transfusions, oedema (knee swelling), wound infection, pain and fever.

**Results:**

THR and TKR orthopaedic surgery resulted in similar changes of coagulation and non-specific inflammatory biomarkers, suggestive of increased coagulation and inflammatory reactions postoperatively. Specifically, THR and TKR surgery resulted in an increase in platelet (*P* = 0.013, THR) and D-dimer (*P* = 0.009, TKR) concentrations. Evidence of increased inflammation was demonstrated by an increase in CRP and ESR (*P* ≤ 0.05, THR and TKR). Four patients received blood transfusions (two THR and two TKR patients), with maximal oedema, pain and aural temperatures peaking between days 1 and 3 postoperatively, for both THR and TKR surgery. None of the patients developed postoperative infections.

**Conclusions:**

The most noticeable changes in biological markers occur during days 1 to 3 postoperatively for both THR and TKR surgery, and these may have an effect on such postoperative clinical outcomes as oedema, pyrexia and pain. This study may assist in understanding the postoperative course following lower limb orthopaedic surgery, and may help clinicians in planning postoperative management and patient care.

## Background

In 2012, the National Joint Register (UK) recorded that 80,314 hip and 84,653 knee replacement (arthroplasty) procedures were undertaken across the country. More than 93% of these procedures were undertaken as a result of osteoarthritis [1].

With an aging society and the raised expectations of our population, joint replacement surgery is likely to increase significantly during the next few years. In an article recently published by Frampton [2], it is estimated that by the year 2030, hip replacement will increase by 157%, while knee replacement will increase by 673%. As a result, it can be appreciated that the development of postoperative complications following joint replacement surgery (for example, infection, systemic inflammatory response syndrome, deep vein thrombosis or haemorrhaging) is likely to increase. Despite considerable progress in the field of orthopaedic surgery, comparing a range of biological markers with the ultimate aim of monitoring or predicting postoperative complications has not yet been extensively researched.

Conventionally in the UK, patients are monitored during the postoperative period by means of a full blood count and urea and electrolytes tests. These tests cannot predict outcome or monitor patients during the postoperative course. However, little evidence is available to demonstrate the effects of lower limb orthopaedic surgery on selective coagulation and inflammatory biomarkers, including various clinical outcome measures.

Changes in leucocyte subpopulations have previously been studied in patients undergoing THR surgery. Specifically, this study involved 12 patients and found leucocytosis, monocytosis, lymphocytopenia and granulocytosis after surgery [3]. It can be appreciated that platelets play a key role during primary haemostasis and aid in the development of blood clotting. Although increased platelet counts have been reported to be dominant contributors to hypercoagulability after injury in surgical intensive care trauma patients, little evidence has been documented with respect to changes in platelet concentrations following lower limb orthopaedic surgery [4]. With regards to the coagulation pathway, fibrin degradation products are formed whenever fibrin is broken down by enzymes (for example, plasmin). D-dimer is an end product derived from plasmin-mediated degradation of cross-linked fibrin clots. D-dimer measurement has proved to be a sensitive marker for the evaluation of disseminated intravascular coagulation (DIC) [5]. In addition, low levels of D-dimer can effectively rule out those patients with a low or a moderate probability of venous thromboembolism, such as deep vein thrombosis or pulmonary embolism [6,7].

C-reactive protein (CRP) is produced in the liver and is a member of the class of acute-phase reactants as its levels rise dramatically during inflammatory processes occurring in the body. The erythrocyte sedimentation rate (ESR) measures the rate at which the red blood cells separate from the plasma. During an inflammatory process, the high proportion of fibrinogen in the blood causes red blood cells to stick to each other. The red cells form stacks, called ‘rouleaux’, which settle more quickly. Margheritini *et al*. [8] performed a study to establish normal values for the ESR and CRP levels after uncomplicated anterior cruciate ligament reconstruction. This investigation involved measurement of ESR and CRP in 45 consecutively treated patients. Blood samples were collected before surgery and on postoperative days 1, 3, 7, 15 and 30. Results from the study demonstrated that both ESR and CRP levels showed a marked increase postoperatively, peaking between the third and seventh postoperative days, with the latter showing a faster return to normal. Findings from this study proposed that measurement of CRP can be used as a more accurate predictor than ESR of postoperative complications. Our study aims to build on previous work and, specifically, to evaluate changes to CRP and ESR values following THR and TKR surgery.

Previous studies have demonstrated the effect of various orthopaedic interventions on surgical complications [9-13]. Despite considerable progress in the field of orthopaedic surgery, surgical complications still exist. Every operation carries a wide range of risks from the most insignificant (for example, low volume blood loss) to the most serious, such as development of deep vein thrombosis, infection and systemic inflammatory response syndrome (including fatal complications). The aim of this study was to test the hypothesis that lower limb orthopaedic surgery results in changes to coagulation, non-specific markers of inflammation (primary objective) and selective clinical outcome measures (secondary objective). It is anticipated that any changes in the measured parameters may provide future direction with respect to therapeutic intervention, which may have an important impact with regards to treatment strategies following orthopaedic trauma.

## Methods

### Subject volunteers

Ethical approval for this pilot study was received from the local research ethics committee (North Wales Central Research Ethics Committee, reference number 05/WNo02/26). Ten volunteers scheduled for either elective THR or TKR surgery were recruited with their informed consent. The test subjects were aged between 58 and 87 years old (mean age = 77 for both THR and TKR), and were all scheduled for elective surgery due to osteoarthritis. Five patients were scheduled for THR (three women and two men) and five patients for TKR (three women and two men).

### Blood samples

Venous blood samples were collected by venepuncture and collected into vacutainers containing EDTA-K_2_, sodium citrate and serum clot activator (Greiner Bio-one, UK). Plasma was obtained by centrifuging whole blood samples at 450*g* for 15 minutes, following which all plasma samples were used for the D-dimer assays.

### Total hip replacement surgery

Prior to surgery, an 18GA cannula (BD Venflon™, Sweden) was inserted into the arm at the antecubital fossa. A venous blood sample was then collected preoperatively, which stood as a baseline measurement for that particular patient. In theatre, patients were prepared for THR surgery by undergoing general anaesthesia. Blood samples were then collected from the arm by means of the cannula following surgery at day 1, 3 and 5 postoperatively. No tourniquet was used during this orthopaedic surgical procedure, although to maintain a bloodless field during surgery, methods such as ligation (clamping and tying off blood vessels) and/or diathermy (heat probe that hyper-coagulates the blood vessels) were employed.

### Total knee replacement surgery

Prior to surgery an 18GA cannula (BD Venflon™, Sweden) was inserted into the arm at the antecubital fossa. A venous blood sample was collected preoperatively, which stood as a baseline measurement for that particular patient. In theatre, patients were prepared for TKR by administering general anaesthesia. Before commencing surgery, a tourniquet was set around the upper thigh and inflated to 315 ± 9.80 mmHg, to ensure a bloodless field prior to surgery. The mean time of ischaemia was 94 ± 7.47 minutes per TKR surgical procedure. Blood samples were collected from the arm by means of the cannula upon the release of the tourniquet at 5 and 15 minutes reperfusion, and on days 1, 3 and 5 postoperatively.

### Measurement of platelet concentration

Following venepuncture, total platelet counts were performed using a Coulter® MicroDiff^18^ blood analyzer (Beckman Coulter, UK).

### Measurement of D-dimer concentration

Levels of the D-dimer parameter were measured using a Mini-Vidas automated immunoassay system that uses enzyme-linked fluorescent assay technology. The Mini-Vidas system and immunoassay kits were obtained from Biomerieux, UK.

### Measurement of C-reactive protein

Highly sensitive CRP measurement was achieved using the Quantex CRP plus kits (Bio-kit, Spain) and involved using a turbidimetric assay as described by Price *et al*. [14]. Measurement was then performed using an ILAB 600 clinical chemistry analyzer (Instrumentation Laboratory, UK).

### Measurement of erythrocyte sedimentation rate

During this study, the Westergren method was employed using kits and racks supplied by Becton Dickinson, UK. The ESR is the rate at which the red blood cells separate from the plasma and fall to the bottom of the tube. The amount of sedimentation was noted after 1 hour, to provide the rate in mm/hr [15].

### Clinical outcome measures

Patients were monitored regularly during the postoperative course to assess for any signs of complication, which included: blood transfusion (the number of ABO/rhesus compatible red cell units); oedema (knee swelling was determined by measuring girth size 10 cm from the tibial tuberosity, the bony prominence on the upper shinbone (tibia), for the TKR surgery study only); Postoperative pain (pain was scored using a point scoring system with 1 indicating no pain; 2, mild pain; 3, moderate pain and 4, severe pain); pyrexia or fever (monitored by measurement of aural (ear) temperatures, with a fever being diagnosed when the temperature was at, or higher than, 37.5°C); infection (wound swabs collected at various time points and incubated for 48 hours on various microbiology plates that included: blood agar, MacConkey agar, an anaerobic medium, and agar selective for *Staphylococcus* and *Streptococcus*.

### Statistical analysis

During this study, all results were presented as either mean or median ± standard deviation (SD). Where data were normally distributed, a repeated measures one-way analysis of variance (ANOVA) between-samples test was employed, adopting a 5% level of significance. Post hoc testing was conducted using Tukey’s test for pairwise comparisons between means. Data that did not comply with normality were analyzed using the Friedman test. Where the Friedman test resulted in statistical significance, subsequent tests were performed using the Wilcoxon test. Statistical significance was accepted for *P* ≤ 0.05.

## Results

### Effect of total hip replacement and total knee replacement surgery on coagulation biomarkers

#### Platelet count

**Figure 1:**
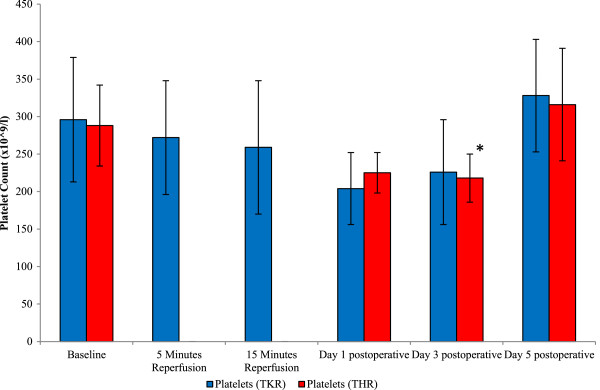
**Effect of THR and TKR surgery on platelet counts.** The points represent mean ± SD. *P* = 0.013 (*n* = 5), as determined by ANOVA (THR). Upon further testing, the pairwise comparison showed significant differences between baseline vs day 3 postoperative (*P = 0.04). ANOVA, analysis of variance; SD, standard deviation; THR, total hip replacement; TKR, total knee replacement.

This parameter was measured as a marker of coagulation activity. With regards to the platelet counts and THR (Figure 1), these decreased from baseline (288 ± 54.87), during day 1 (225 ± 27.15) and day 3 (218 ± 32.0) postoperatively. By day 5 postoperatively the platelet counts had increased to levels above that of basal values (316 ± 75.54), (*P* = 0.013 as determined by ANOVA). Upon further analysis, pairwise comparison testing showed significant differences between baseline and day 3 postoperative (*P* = 0.04). With regards to the platelet counts and TKR (Figure 1), these decreased from baseline (296 ± 83.97), during 5 (272 ± 76.64), 15 (259 ± 89.67) minutes reperfusion and day 1 postoperative (204 ± 48.23). By day 5 (328 ± 75.70) postoperatively the platelet counts had increased to levels above that of basal values, although these did not reach statistical significance (*P* = 0.06, as determined by the Friedman test).

#### D-dimer concentration

**Figure 2:**
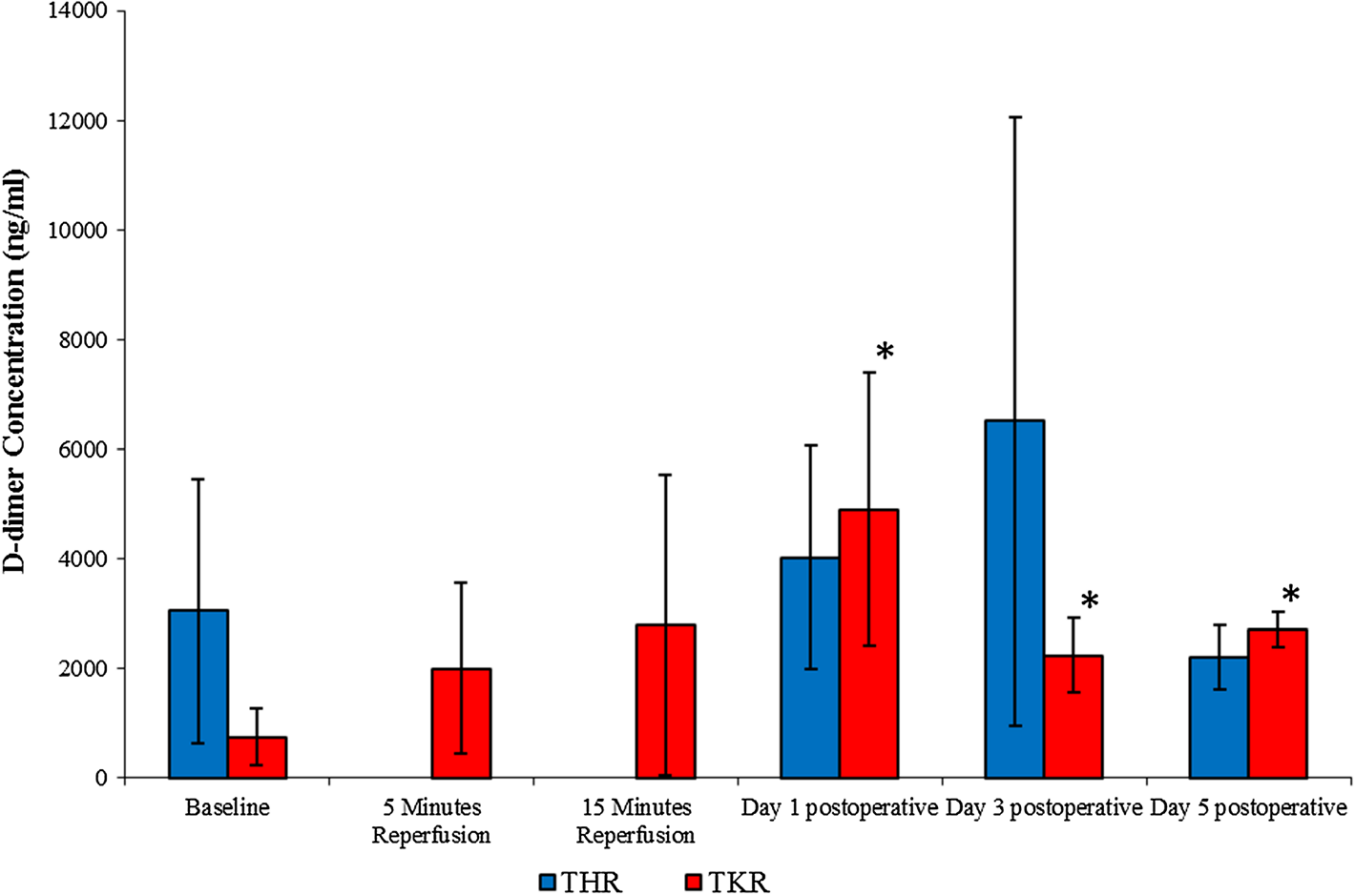
**Effect of THR and TKR surgery on D-dimer concentration.** The points represent median ± SD, *P* = 0.009 following TKR surgery, as determined by the Friedman test. *P ≤ 0.05 baseline vs day 1, day 3 and day 5 postoperative for TKR surgery, as determined by the Wilcoxon test, *n* = 10. ANOVA, analysis of variance; SD, standard deviation; THR, total hip replacement; TKR, total knee replacement.

This parameter was measured as a marker of coagulation activity (Figure 2). Although no significant changes were observed in the D-dimer concentration (ng/ml) following THR surgery (*P* = 0.323, as determined by the Friedman test), a trend of increasing D-dimer concentration from baseline (3050.36 ± 2402.66), during day 1 (4026.01 ± 2030.04), and peaking at day 3 postoperative, was seen (6510.60 ± 5548.89). At day 5 (2207.56 ± 583.46) postoperative the D-dimer concentration decreased to levels lower than that of basal values.

With regards to TKR surgery, significant changes were observed in D-dimer concentrations (*P* = 0.009, as determined by the Friedman test). D-dimer concentrations increased from baseline (754.59 ± 517.54), during 5 (2005.79 ± 1565.17) and 15 (2789.55 ± 2747.24) minutes reperfusion, and peaked at day 1 (4899.40 ± 2490.35) postoperatively. The concentration decreased at day 3 (2243.83 ± 678.45) and day 5 (2709.85 ± 323.29) postoperatively, although it remained at a higher level to that of basal values (three-fold). Upon further analysis, the Wilcoxon test showed significant differences between baseline and day 1, day 3 and day 5 postoperatively (*P* ≤ 0.05).

### Effect of total hip replacement and total knee replacement on non-specific inflammatory markers

#### Erythrocyte sedimentation rate

**Figure 3:**
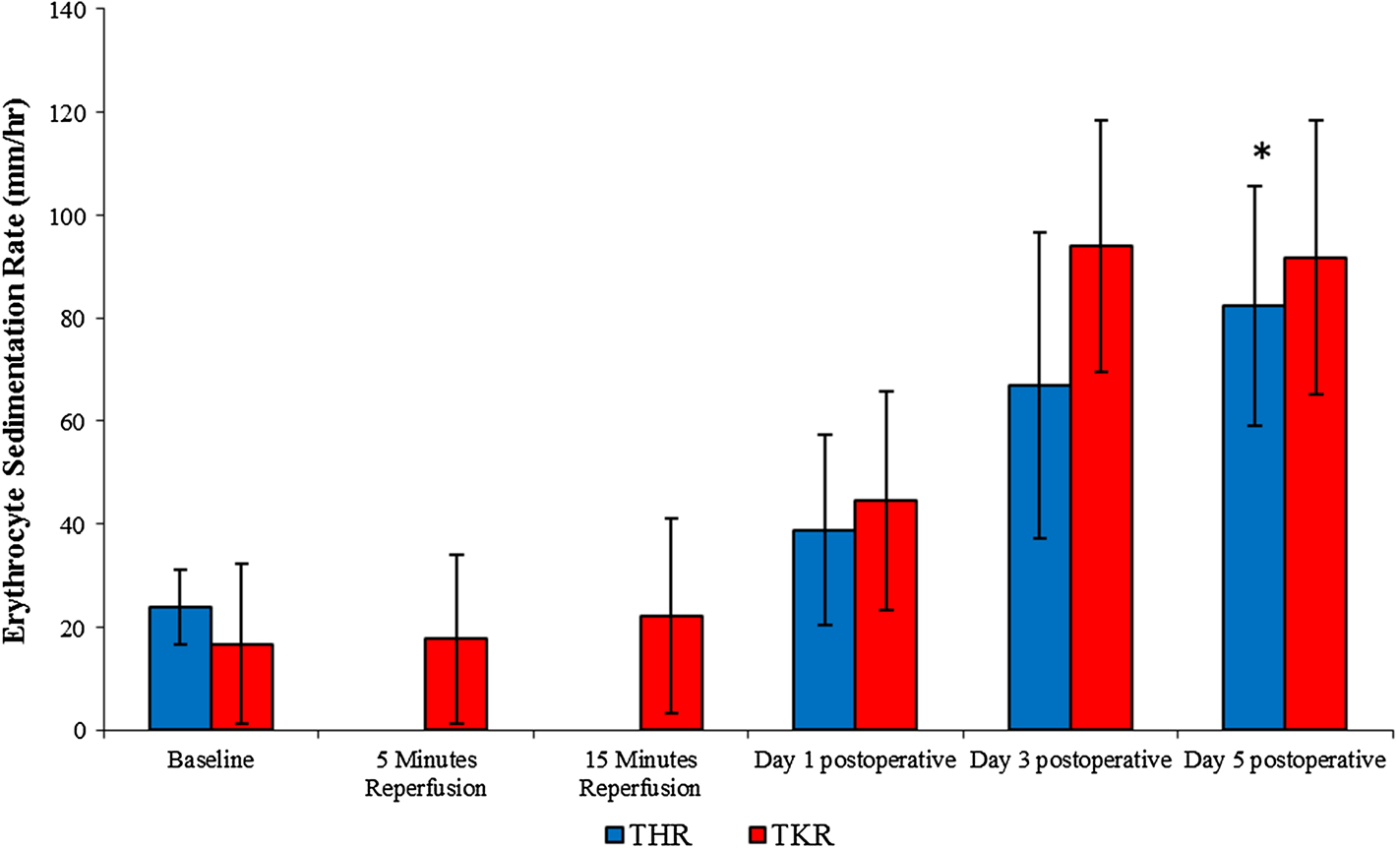
**Effect of THR and TKR surgery on ESR.** The points represent mean or median?±?SD, *P*?=?0.05 for both THR and TKR surgery, as determined by ANOVA and the Friedman tests, respectively. Baseline vs day 5 postoperative for THR (*P?=?0.027), as determined by pairwise comparison testing, *n*?=?10. ANOVA, analysis of variance; SD, standard deviation; THR, total hip replacement; TKR, total knee replacement.

Samples were collected prior to and following THR and TKR surgery, and the ESRs were analyzed using the Westergren method (mm/hr). This parameter was measured as a marker of non-specific inflammation (Figure 3). Following THR surgery, significant changes were seen in the ESRs (*P* = 0.001, as determined by ANOVA). ESRs increased from baseline (23.80 ± 7.22), and at day 1 (38.80 + 18.43) and day 3 (67.00 ± 29.69), peaking at day 5 (82.40 ± 23.30) postoperatively. Upon further analysis, pairwise comparison testing showed significant differences between baseline and day 5 (*P* = 0.027) postoperatively.

With regards to TKR surgery, significant changes were observed in ESRs (*P* = 0.002, as determined by the Friedman test). These values increased from baseline (16.75 ± 15.69), during 5 (17.75 ± 16.42) and 15 (22.00 ± 18.92) minutes reperfusion and at day 1 (44.50 ± 21.29) postoperative, peaking at day 3 (94.00 ± 24.49) postoperatively. ESRs decreased very slightly at day 5 (91.75 ± 26.51) postoperatively, and remained at a higher level than those of basal values (five-fold). Upon further analysis, the Wilcoxon test showed no significant differences between the measured parameters.

#### C-reactive protein

**Figure 4:**
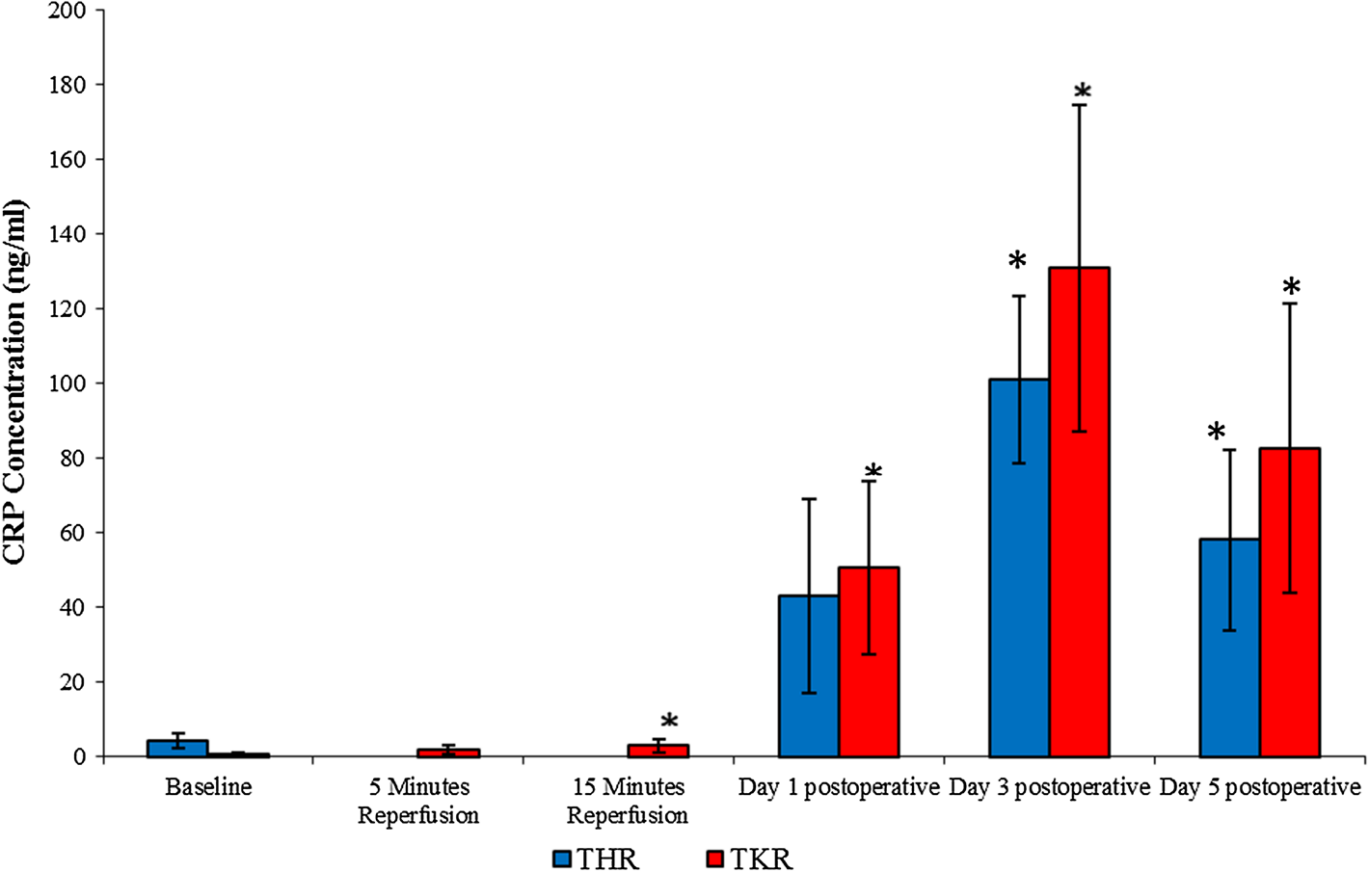
**Effect of THR and TKR surgery on CRP concentration.** The points represent mean or median ± SD, *P* ≤ 0.001 for both THR and TKR surgery, as determined by ANOVA and the Friedman test respectively. *P ≤ 0.05 Baseline vs day 3 and day 5 postoperative for THR (pairwise comparison), and baseline vs 15 minutes reperfusion, day 1, day 3 and day 5 postoperatively for TKR surgery (Wilcoxon), *n* = 10. ANOVA, analysis of variance; SD, standard deviation; THR, total hip replacement; TKR, total knee replacement.

The results are expressed as ng/ml and represent the changes in CRP concentration (a non-specific marker of inflammation) following THR and TKR surgery (Figure 4). Following THR surgery significant changes were seen in CRP concentrations (*P* ≤ 0.001, as determined by ANOVA). CRP concentrations increased from baseline (4.18 ± 2.03), at day 1 (43.14 ± 25.83) and peaking at day 3 (101.24 ± 22.48) postoperatively. CRP concentration decreased towards basal levels at day 5 (58.22 ± 24.28) postoperatively, although remained at a higher level to those of basal values (14 fold). Upon further analysis, pairwise comparison testing showed significant differences between baseline *vs* day 3 (*P* = 0.004) and day 5 (*P* = 0.048) postoperatively.

With regards to TKR surgery, significant changes were observed in CRP concentrations (*P* ≤ 0.001, as determined by the Friedman test). CRP concentrations increased from baseline (0.62 ± 0.50), during 5 (1.76 ± 1.24) and 15 (3.06 ± 1.78) minutes reperfusion and at day 1 (50.64 ± 23.19) postoperative, peaking at day 3 (131.26 ±43.78) postoperatively. The CRP concentration decreased at day 5 (82.68 ± 38.86) postoperatively, although it remained at a higher level than those of basal values (136 fold). Upon further analysis, the Wilcoxon test showed significant differences between baseline and 15 minutes reperfusion, day 1, day 3 and day 5 postoperatively (*P* ≤ 0.05).

### Effect of THR and TKR on clinical outcome measures

#### Blood transfusion

**Figure 5:**
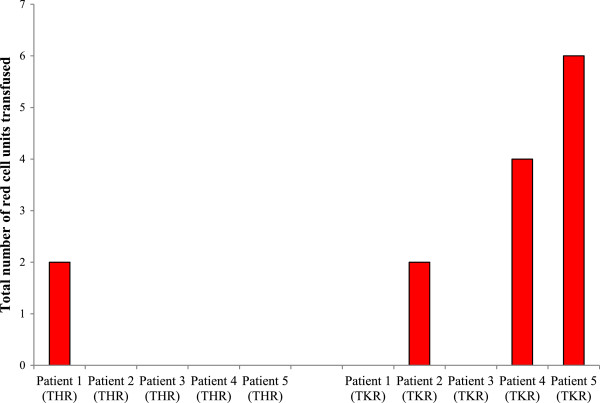
**Effect of THR and TKR surgery on total red cell units transfused.** Transfusion of ABO and rhesus compatible red blood cells, *n* = 10. THR, total hip replacement; TKR, total knee replacement.

Patients received transfusions of standard National Health Service Blood and Transplant (UK) ABO/rhesus compatible donor red blood cell units following surgery at the orthopaedic surgeon’s discretion. Following THR surgery, patient 1 received two units of cross-matched red blood cells (Figure 5). Patients 2 to 5 did not require any blood transfusion following THR surgery. With regard to TKR surgery, patients 1 and 3 required no blood transfusions. Patient 2 received two units of cross-matched blood following surgery. Patients 4 and 5 received 4 and 6 units of cross-matched blood, respectively. None of the patients received platelet transfusions.

### Oedema (total knee replacement only)

**Figure 6:**
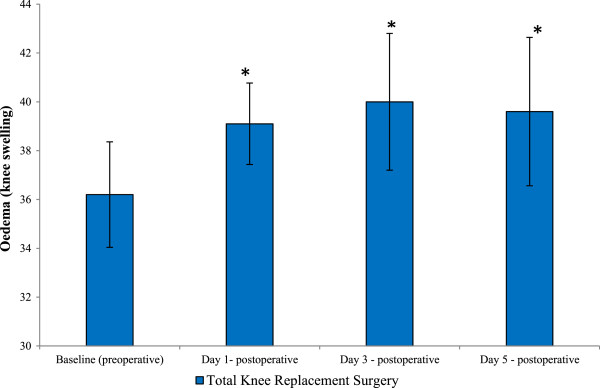
**Effect of THR and TKR surgery on oedema.** Measurement of fluid retention (knee swelling) was performed by measuring girth size 10 cm from the tibial tuberosity (the bony prominence on the upper shinbone (tibia)). The points represent mean ± SD, *P* = 0.003 as determined by ANOVA. Baseline vs days 1 (*P = 0.002), 3 (*P = 0.004) and 5 (*P = 0.045) postoperative, *n* = 5 (five TKR patients). ANOVA, analysis of variance; SD, standard deviation; THR, total hip replacement; TKR, total knee replacement.

Postoperative oedema (knee swelling) was assessed by measuring girth size 10 cm from the tibial tuberosity (the bony prominence on the upper shinbone) and was performed on TKR patients only (Figure 6). Following TKR surgery, oedema was demonstrated to increase significantly (*P* = 0.003, as determined by ANOVA). Oedema in TKR patients increased from baseline (36.2 cm), at day 1 (39.1 cm), peaking at day 3 postoperative (40 cm). By day 5, postoperative fluid retention levels had reduced slightly (39.6 cm), although they still remained higher than those of basal values. Upon further testing by Tukey’s pairwise comparisons, significant differences between baseline and day 1 postoperative (*P* = 0.002), day 3 postoperative (*P* = 0.004) and day 5 postoperative (*P* = 0.045) were observed.

### Postoperative pain

**Figure 7:**
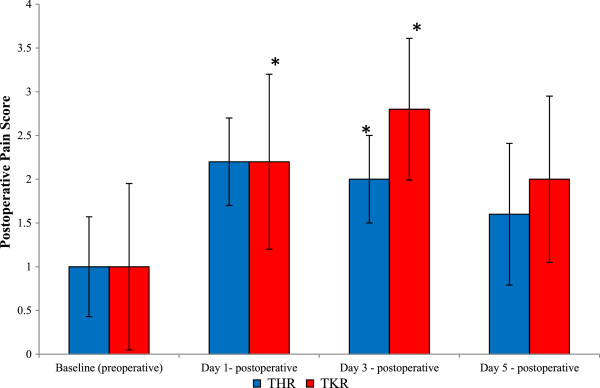
**Effect of THR and TKR surgery on postoperative pain.** Postoperative pain was measured by a point scoring system with 1 indicating no pain; 2, mild pain; 3, moderate pain and 4, severe pain. The points represent median ± SD, as determined by the Friedman test (*P* = 0.020 THR, *P* = 0.012 TKR). Baseline vs day3 (*P = 0.025) postoperative TKR; Baseline vs days 1 and 3 (*P = 0.025 and *P = 0.034, respectively) postoperative TKR, *n* = 10. SD, standard deviation; THR, total hip replacement; TKR, total knee replacement.

It can be appreciated that the assessment of pain is difficult, owing to its subjective nature. However, pain was monitored during this study and was assessed by a point scoring system routinely used by the nursing and medical staff at the North Wales Medical Centre (Figure 7). Following THR surgery postoperative pain significantly increased (*P* = 0.020, as determined by the Friedman test). Pain increased from baseline (pain score, 1) and peaked at day 1 (mean pain score, 2.2) postoperative, which was demonstrated to be mild pain. By day 3 (mean pain score, 2) and day 5 (mean pain score, 1.6) postoperative, the mean pain scores decreased although still remained higher to those of basal values. Upon further testing using the Wilcoxon test, there were significant differences between baseline *vs* day 3 postoperative (*P* = 0.025).

Following TKR surgery postoperative pain significantly increased (*P* = 0.012, as determined by the Friedman test). Pain increased from baseline (mean pain score, 1) and during day 1 (mean pain score, 2.2), and peaked at day 3 (mean pain score, 2.8) postoperative, when pain was scored as mild to moderate. By day 5 (mean pain score, 2) postoperative, the mean pain scores decreased although they still remained higher than those of basal values, with patients experiencing mild pain overall. Upon further testing using the Wilcoxon test, there were significant differences between baseline vs day 1 postoperative (*P* = 0.025) and day 3 postoperative (*P* = 0.034).

### Aural temperature

**Figure 8:**
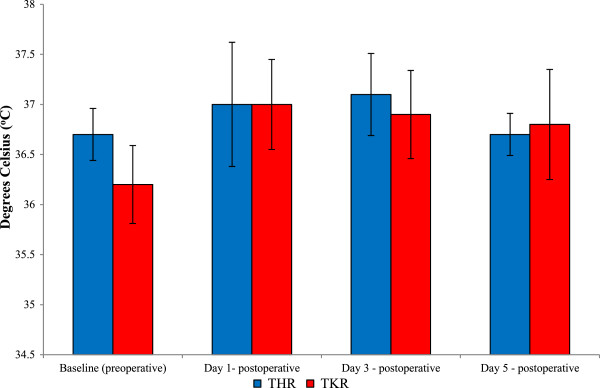
**Effect of THR and TKR surgery on aural temperature.** Pyrexia (fever) was monitored by measurement of aural temperatures, with pyrexia diagnosed when the temperature was at, or higher than, 37.5°C. The points represent mean ± SD, *P* ≥ 0.05 for THR and TKR, *n* = 10. SD, standard deviation; THR, total hip replacement; TKR, total knee replacement.

Postoperative pyrexia was diagnosed when the aural body temperature was at, or higher than 37.5°C by means of an auricular thermometer (Figure 8). The value of 36.8°C ± 0.7°C was considered to be the normal aural temperature. Following THR surgery the mean aural temperature increased from baseline (36.7°C), during day 1 postoperative (37.0°C) and peaking at day 3 (37.1°C) postoperatively. By day 5 postoperative the aural temperatures had returned towards basal values (36.7°C).

Following TKR surgery, the mean aural temperature increased from baseline (36.2°C) and peaked at day 1 (37.0°C) postoperatively. By day 3 (36.9°C) and day 5 (36.8°C) postoperative, the aural temperatures had returned towards basal values. Although a trend of increasing pyrexia following both THR and TKR orthopaedic surgeries was observed, these did not reach statistical significance (*P* = 0.372 THR surgery, as determined by the Friedman test, and *P* = 0.146 TKR, as determined by ANOVA).

During this study four patients (two THR and two TKR) developed postoperative fevers (individual patient data not shown).

### Wound infection

Wound swabs were collected and assessed for evidence of infection at the site of surgery (Table 1). They were collected before and after THR and TKR surgery (day 3 postoperative), and were assessed at the Microbiology Laboratory at Glan Clwyd Hospital, North Wales (UK). They were incubated for 48 hour periods on blood agar, MacConkey agar, anaerobic medium, and *Staphylococcus* and *Streptococcus* selective agar plates and were all negative.

**Table 1:** Effect of THR and TKR surgery on wound infection

	**THR (preoperatively)**	**THR (day 3 postoperatively)**	**TKR (preoperatively)**	**TKR (day 3 postoperatively)**
Blood agar	Negative	Negative	Negative	Negative
MacConkey	Negative	Negative	Negative	Negative
Anaerobic medium	Negative	Negative	Negative	Negative
*Staphylococcus* selective agar	Negative	Negative	Negative	Negative
*Streptococcus* selective agar	Negative	Negative	Negative	Negative

Wound swabs were collected and assessed for evidence of infection at the site of surgery. Swabs were collected pre and post THR and TKR surgery (day 3 postoperative). Swabs were incubated for 48 hour periods on blood agar, MacConkey agar, anaerobic medium and agar selective for *Staphylococcus* and *Streptococcus*.

## Discussion

An important aspect of this study was the evaluation of selective biomarkers and clinical outcome measures, which would provide a better understanding of the postoperative course following elective lower limb orthopaedic surgery.

Results from the study demonstrated evidence of decreasing platelet concentration following THR surgery. This surgery resulted in a decrease in platelet counts (day 1 and day 3 postoperatively), with the platelet concentrations increasing to that above basal values (preoperatively) at day 5 postoperatively (*P* ≤ 0.05). Similar trends were observed following TKR surgery. The results obtained during this study build on previous work by Høgevold *et al.*[3], which provided evidence of leucocytosis following THR surgery, and provides further evidence of thrombocytic involvement during the postoperative course. Our findings also complement work by Harr *et al.*[4] that demonstrated increased platelet counts and subsequent hypercoagulability in other surgical settings. It may therefore be inferred that following long-bone surgical intervention there is a systemic response resulting in leucocytosis and thrombocytosis. These changes are possibly due to increased bone marrow turnover, which has resulted from THR and TKR surgery procedures, postoperative wound and tissue repair or, probably, a combination of these contributing factors.

Concentrations of D-dimer were measured as a marker of coagulation activity. Specifically, D-dimer concentration in the plasma was affected following TKR (*P* ≤ 0.05) surgery. This increased from baseline (preoperatively) up to day 1 postoperatively. Changes in D-dimer concentrations demonstrate an increased coagulation activity following surgery, with a possible explanation for these changes being that TKR surgery has an intrinsic effect on normal homeostasis, resulting in changes in the coagulation pathways; this may lead to postoperative complications, such as deep vein thrombosis. Muñoz *et al.*[16] have reported that D-dimer levels increased on day 1 post TKR surgery and remained elevated at day 30 postoperative. They reported that increased D-dimer levels following TKR surgery do induce changes in coagulation activity, with these changes not being modified by either allogenic, unwashed drainage or no blood transfusion. In agreement with Muñoz *et al.*[16], this study demonstrates evidence of increased coagulation activity following TKR surgery, and provides further evidence that similar yet insignificant changes were observed following THR surgery.

This study assessed changes in inflammatory markers following THR and TKR surgery. It was demonstrated that there was a significant effect of surgery on the CRP level and ESR, which were indicative of an increased systemic inflammatory response during the acute phase post-surgery. The results are in accordance with Margheritini *et al*. [8] who demonstrated an increase in both CRP levels and ESR (peaking between day 3 and day 7 post-surgery), following uncomplicated anterior cruciate ligament reconstruction. This study illustrates that similar patterns were observed, demonstrating an increased inflammatory response following THR and TKR surgery, peaking at day 3 postoperative with respect to CRP levels. However, ESR values peaked at day 5 postoperative following THR and TKR. With respect to measurement of both these parameters, which are considered to be sensitive non-specific markers of inflammation, it was demonstrated that both tests were effective methods of measuring acute inflammatory response post-surgery. However, it may be considered that measuring CRP is more efficient than ESR, in that CRP levels increased dramatically up to day 3 postoperative, showing a higher fold increase, whereas the ESR displayed a gradual increase during this period. A possible explanation for the increase in ESR is that rouleaux formation and red cell clumping are mainly controlled by the concentrations of fibrinogen and other acute-phase proteins, such as CRP, which may play an intrinsic role that affects the ESR results observed postoperatively. This suggests that CRP levels subsequently affect ESR. Furthermore, the finding that the increase for CRP was greater than that of ESR suggests that CRP concentration is a more sensitive marker of inflammation. This was supported by the finding that CRP levels dropped at day 5, whereas ESRs remained high at this time point. Our results are in general accord with White *et al*. [17], who demonstrated an increase in CRP on the second day post TKR; our study demonstrated that CRP levels peaked at day 3 postoperatively (following THR and TKR surgery), with the CRP values following TKR surgery being slightly greater than those of THR at this time. Results from this study also complement findings published by Orrego *et al*. [18], who demonstrated that CRP levels fluctuated after various elective orthopaedic surgical procedures, with maximum values being observed between the second and third postoperative day. The elevated levels of CRP observed may be due to the release of pro-inflammatory cytokines, such as interleukin-1 and interleukin-6 from the activated cells in response to surgery and inflammation [19,20].

With regards to the clinical outcome measures, various parameters were measured prior to and following THR and TKR surgery. Assessment of the number of red cell units administered to each patient following THR and TKR surgery demonstrated that patients scheduled for TKR surgery required more blood (packed red cells) postoperatively than THR patients. In agreement with Sakić and Sakić [9], the present study demonstrates that the number of red cell units transfused per patient (if any) is determined based on routine clinical and laboratory findings postoperatively, as performed by consultants, medical officers and nursing staff. However, it is important to note that for the patients who required blood transfusions during these clinical studies, the introduction of foreign material, in the form of packed red blood cells, to the peripheral blood system may have an effect on the biological markers measured and the outcome of some of the results obtained. Nevertheless, to fully appreciate the systemic complication that blood transfusion may have had on the biological markers measured in this study, further independent studies would need to be performed, to compare directly the effects of surgery on the various biological markers for those patients who did and did not receive blood transfusions. During this pilot study, the measured parameters did not differ between patients receiving blood and those without.

Measurement of oedema (swelling of the knee) following TKR surgery was performed by measuring girth size 10 cm from the tibial tuberosity. A previous study has demonstrated that the loss of knee-extension strength following total knee arthroplasty is related to increased knee swelling [21]. In agreement with others [21], results from our investigations demonstrated that, following TKR surgery, oedema was most prevalent at day 3 postoperative (*P* ≤ 0.05). Interpretation of these results may suggest that at day 3 postoperative the inflammatory response during the acute phase post-surgery may have been at its highest, resulting in local oedema around the surgical area. With respect to oedema, it can be appreciated that anything that increases osmotic pressure outside blood vessels (for example inflammation), or reduces osmotic pressure in the blood (states of low plasma osmolality) will cause oedema, and this may suggest that some of the inflammatory markers measured during these studies could relate to this.

Postoperative pain and management has been reported by others previously [12], and during this study analgesics were prescribed as standard treatment postoperative to minimize any pain and discomfort following surgery. However, results obtained from this study demonstrated that, following THR and TKR, most pain was experienced at day 3 regardless of prescribed analgesics to help minimize any pain. Postoperative pyrexia (fever) was diagnosed when measurement of the aural temperature was at, or higher than 37.5°C. During this study four patients (two THR and two TKR) developed postoperative fevers (data not shown), although there was no overall significant temperature increase following THR and TKR surgery. Although, previous studies have been performed to investigate fevers and orthopaedic surgery [10-13], this study confirmed that developing fevers post-surgery is still a postoperative complication to be considered and treated by medical officers and surgeons. Interestingly, the fevers that developed during the current study occurred without any infection, which may suggest that this may be a normal physiological response to surgery in certain patients.

With regards to postoperative infection, none of the patients involved during this study developed wound infection. Therefore, infection probably does not explain the changes in biological markers that have been found. The orthopaedic surgeons involved in the study (and as part of standard practice) routinely administered antibiotics to all their patients pre-surgery and post-surgery to minimize any risk of infection and the potential of developing postoperative sepsis (a systemic response to infection).

Although monitoring patients to assess for any signs of postoperative complications was performed, it was felt that in order to achieve a more accurate understanding of the effects of THR and TKR surgery on these clinical parameters, a more detailed clinical assessment would be required. It should also be appreciated that for those patients requiring TKR surgery, an application of a tourniquet was employed. TKR patients were subjected to long periods of ischaemia (94 ± 7.47 minutes), with tourniquet pressure being set as a standard up to 350 mmHg. We have previously shown the effects of ischaemia and reperfusion on non-surgical models, but the main purpose of this study was to determine the effects of THR and TKR surgery on various biological markers, and not to develop a clinical model of ischaemia-reperfusion injury [22].

Although it was interesting to investigate clinical outcome measures during this study, the primary objective was to investigate the effects of surgery on various biological markers. Despite the fact that some interesting clinical outcome observations were made as a secondary measure, further studies would have to be performed to fully explore any relationships between the biological markers measured and the clinical outcome measures assessed. However, in principle this study provides a sound platform to further investigate these possibilities and to try to determine any links between the laboratory data and those obtained from the clinical outcome measures.

In summary, it is proposed that changes in the measured parameters during this study may not be due to a single factor, but due to a number of factors that may result following surgery such as tissue damage, wound repair, introduction of foreign material (for example, prosthesis or red blood cells via blood transfusion) and ischaemia-reperfusion injury during joint replacement surgery.

## Conclusions

It appears that changes in biological markers following THR and TKR surgery followed a similar trend. Most noticeable changes in biological markers occur during days 1 to 3 postoperatively for both THR and TKR surgery, and these may have an effect on some of the clinical outcomes, such as oedema, pyrexia and postoperative pain that develop post-surgery. This study may therefore have clinical implications for understanding the postoperative course following lower limb orthopaedic surgery, and may help clinicians in planning their postoperative management and patient care. The study may also provide an opportunity to highlight potential points of therapeutic intervention, which ultimately promote patient recovery following major orthopaedic surgery.

## Abbreviations

ANOVA: Analysis of variance; CRP: C-reactive protein; ESR: Erythrocyte sedimentation rate; SD: Standard deviation; THR: Total hip replacement (arthroplasty); TKR: Total knee replacement (arthroplasty).

## Competing interests

The authors declare that they have no competing interests.

## Authors’ contributions

SFH carried out the coagulation investigations (platelet and D-dimer assays). BDH assessed the inflammatory response (CRP and ESR). SSB participated in the design and collection of the blood sampling procedure. DRE and SSB advised on the clinical implications. SFH and JFM supervised the study, participated in its design and coordination and drafted the manuscript. All authors read and approved the final manuscript.
